# Addressing the Wicked Problem of Obesity through Planning and Policies

**DOI:** 10.1371/journal.pmed.1001475

**Published:** 2013-06-25

**Authors:** The PLOS Medicine Editors

## Abstract

In the June editorial, the *PLOS Medicine* Editors highlight two papers and associated perspectives published recently in *PLOS Medicine* on the link between physical activity and noncommunicable diseases, and discuss possible approaches to the “wicked” problem of inactivity.

*Please see later in the article for the Editors' Summary*

In the two cities where PLOS has its main offices, there seems to be clear recognition of the need for modern transport policies to include transit not just by vehicle (cars, buses), but also by walking and cycling. In Cambridge, UK, for example 32% of commuters cycle to work and 52% of adults cycle at least once a week [1]. In San Francisco campaigners are aiming for 20% of all journeys to be by bike by 2020 [2].

However, the relatively high levels of physical activity in these cities are far from the norm in the more developed world. Much lower levels are more usual; one reason why is that changing behaviors to increase activity is an example of a “wicked” problem in health policy. Wicked problems are not evil but rather are “difficult or impossible to solve because of incomplete, contradictory, and changing requirements that are often difficult to recognize” [3]. To give just two characteristics of a wicked problem: the problem itself is hard to define, and the problem can itself be considered to be a symptom of other problem(s) – for example, in the case of inactivity the fact that town planning has traditionally been dominated by the needs of the car. However, the need to address physical inactivity is urgent: the World Health Organization now ranks physical inactivity as the fourth leading global risk factor for mortality, which in turn is a major cause of non-communicable diseases (NCDs), the leading cause of death globally [4]

Two papers recently published in *PLOS Medicine* examine different aspects of physical activity in the context of public health. The first, by Christopher Millet and colleagues [5], documents the associations between active travel to work and overweight, hypertension, and diabetes in India. The authors compared modes of travel to work—walking, bicycling, or going by public transport versus travel by private transport—and found that active travel (which includes public transport) was associated with health benefits; specifically, those bicycling were significantly less likely to have hypertension or diabetes than those who went to work by private transport. This analysis not only adds to a growing body of evidence of the long-term benefits of exercise, but is particularly valuable as it indicates that exercise incorporated into usual daily activities, such as commuting to work, can be associated with substantial benefit.

This finding is particularly timely in light of the second paper published recently, a systematic policy review by Patrick Kolsteren and colleagues [6] demonstrating that in low- and middle-income countries (LMICs), where the burden of NCDs is high and rising, the gap between policy and burden is substantial. In the review, although NCD strategies were found for 47% (54/116) of LMICs, specific actions to promote healthier diets and physical activity were present in only a minority. It is also notable that the lack of policies is in direct contradiction to a specific global commitment made in 2004 at the World Health Assembly for actions that addressed lifestyle, diet, and physical activity [7]. Furthermore, even in this digital age the NCD strategies that had been written are not easy to find or access, which raises the question of whether the policies had any purpose beyond box-ticking exercises. The authors of the systematic review suggest that “An open access, full text global repository of initiatives and policies to address NCDs would be a great step forward. It could also contribute to global leadership and shared accountability in the global fight against NCDs.” They go on to offer to organize such an open-access repository and suggest that such a database could be linked to surveillance data on NCD risk. It could also go further and link out to the published literature. Such a resource could become a powerful tool for identifying gaps in evidence, and potentially a resource against which to map implementation of policies—something that is itself poorly documented.

These are critical times in the fight against NCDs and its associated risk factors, but whether any concerted action will happen remains to be seen and may depend on how two specific issues are handled. The first is how to manage conflicting interests. As *PLOS Medicine*'s Big Food series [8] highlighted last year, the food industry is tremendously successful in terms of lobbying governments. Similarly, urban planning was and is influenced by other vested interests, the most notorious example being the lobbying by General Motors in the US to remove streetcars in Los Angeles in the early 20^th^ century [9]. Governments and others who develop policy will need to develop robust mechanisms for ensuring that those with vested interests do not drive policy here and be prepared to stand up to intense lobbying.

Secondly, there is a need to understand much better what policies work and what do not. As David Stuckler and Sanjay Basu say [10] in a Perspective that accompanies Kolsteren and colleagues' paper, “A clear next step is to extend global monitoring systems, such as the WHO Global Monitoring Framework, to cover NCDs and the policies that aim to reduce them.” Highlighting the privately organized Global Burden of Disease Project, they note that it has helped to identify the increase in NCDs but that “many statistics from that project are ‘imputed’ estimates, meaning that little or no on-the-ground data are available from many countries…” They go onto note the critical need to understand what actually works. Most of the evidence in public health has come from careful epidemiological studies, which can only show association, not causation. But most policies are not even studied retrospectively and even fewer are tested prospectively. But there is no reason why the design of clinical trials can't be applied to policies. This is the premise long advocated by Ben Goldacre and others, and in the UK a Cabinet Office initiative called the Behavioral Insights Team (BIT) [11] is doing just that [12].

The time is now right for many initiatives to come together in the global push around public health policies for inactivity and other risk factors for NCDs. With at least one government (of the UK) now receptive to trials of policies, and a recognition of the need for the results of the studies as well as the policies to be openly available, there is an opportunity not only to bring hard evidence to bear on policies but also to exploit the collaborative nature of the internet to enable translation of policies into action.
